# Preparation and Characterization of Pullulan-Based Packaging Paper for Fruit Preservation

**DOI:** 10.3390/molecules29061394

**Published:** 2024-03-21

**Authors:** Hang Dong, Zhongjian Tian

**Affiliations:** State Key Laboratory of Biobased Material and Green Papermaking, Qilu University of Technology, Shandong Academy of Sciences, Jinan 250353, China; dh19971204@163.com

**Keywords:** pullulan-based paper, antibacterial, antioxidant, fruit preservation, shelf life

## Abstract

Improving the shelf lives of fruits is challenging. The biodegradable polysaccharide pullulan exhibits excellent film-forming ability, gas barrier performance, and natural decomposability, making it an optimal material for fruit preservation. To overcome problems of high cost and film porosity of existing packaging technologies, we aimed to develop pullulan-based packaging paper to enhance the shelf lives of fruits. A thin paper coating comprising a mixture of 15 wt.% pullulan solution at various standard viscosities (75.6, 77.8, and 108.5 mPa·s) with tea polyphenols (15:2) and/or vitamin C (150:1) improved the oxygen transmission rate (120–160 cm^3^ m^−2^·24 h·0.1 MPa), water vapor transmission rate (<5.44 g·mm^−1^ m^−2^·h·kPa), maximum free radical clearance rate (>87%), and antibacterial properties of base packaging paper. Grapes wrapped with these pullulan-based papers exhibited less weight loss (>4.41%) and improved hardness (>16.4%) after 10 days of storage compared to those of control grapes (wrapped in untreated/base paper). Grapes wrapped with pullulan-based paper had >12.6 wt.% total soluble solids, >1.5 mg/g soluble protein, >0.44 wt.% titratable acidity, and ≥4.5 mg 100 g^−1^ ascorbic acid. Thus, pullulan-based paper may prolong the shelf life of grapes with operational convenience, offering immense value for fruit preservation.

## 1. Introduction

Preserving postharvest fruits has been a long-standing challenge owing to factors such as the presence of microorganisms (bacteria, fungi, and molds), metabolism of fruits, and evaporation. The contents of total soluble solids (TSSs), vitamin C (Vc), proteins, titratable acids (TAs), and other components in fruits decrease rapidly over time [1], impacting the nutritional value and quality and causing rot and deterioration. Every year, approximately 12 million tons of fruit are lost globally owing to inadequate storage and transportation facilities. Implementing suitable storage techniques would effectively mitigate fruit waste. Various techniques are currently employed to preserve fruits; these include plastic film packaging, refrigeration at low temperatures [2], gas preservation, and use of chemical biocides. However, most preservation methods have certain limitations and disadvantages. For instance, plastic films are typically made from petroleum-based materials that do not degrade readily in the environment [3], leading to environmental pollution and resource waste. Cold storage requires specific environmental conditions, specialized equipment, and high energy consumption; moreover, different fruits need to be stored at different temperatures, which can lower the quality of some fruits owing to an unsuitable temperature. Chemical biocides tend to accumulate in food products, creating uncertainty regarding their safety [4].

Coatings can extend the shelf lives of fruits, prevent nutrient loss, and maintain their original color. For example, Chen et al. [5] effectively extended the preservation of Newhall navel oranges using a new coating made of 1.5% chitosan from hairy fig (*Ficus hirta* Vahl.) fruit extract, resulting in a low decay rate (5.2%) and reduced weight loss (5.16%). In addition, Sultan et al. [6] utilized a chitosan–beeswax film to protect postharvest Le Conte pears, which displayed strong self-healing abilities ranging from 86.7% to 96.3%. Moreover, the chitosan–beeswax/pollen-grain film demonstrated a 50% lower water vapor transmission rate (WVTR) than did the chitosan-only film, the elongation at break decreased from 35.81% to 14.09%, and all coated fruits exhibited reduced weight loss, decay, and softening rates. Cheng et al. [7] used a chitosan–catechin coating to prolong the preservation time of satsuma oranges; integrating catechin into chitosan films improved the freshness and prolonged the duration for which the fruits could be preserved. Liu et al. [8] investigated the regulatory action of an edible coating (preharvest treatment) on the postharvest quality of blueberries in terms of their physiological, biochemical, and organoleptic characteristics. Chi et al. [9] developed a fruit coating comprising locust bean gum, carboxycellulose nanocrystal, and ZnO blended with bayberry tannins, which exhibited good antioxidant, antibacterial, and ultraviolet light-shielding properties. Zhou et al. [10] created a fruit-coating material with multiple functions using quaternized catechol-functionalized chitosan grafted with 2,3-epoxypropyl trimethyl ammonium chloride and 3,4-dihydroxy benzaldehyde, which demonstrated considerable potential in terms of fruit preservation at the industrial level. Issa et al. [11] prepared a sweet potato starch-based nanocomposite film activated with thyme essential oil. This composite film significantly reduced the *Escherichia coli* and *Salmonella typhi* counts on young fresh spinach leaves, which exhibited better odor, color, and appearance than those of control spinach leaves. Min et al. [12] prepared pullulan/polyvinyl alcohol nanofibers incorporated in thymol-loaded porphyrin metal-organic framework nanoparticles (THY@ PCN-224 NPs) for food packaging. This film killed *E. coli* and *Staphylococcus aureus* by >96%, and strawberries were stored for an increased period of time (at least 3 days) and maintained a high level of freshness when using this packaging material. Kang et al. [13] developed polyphenol-loaded pullulan/trehalose composite films able to inhibit *E. coli* and *S. aureus* growth rates by >90% after 8 h, effectively extending the shelf life of apple cuttings and reducing weight loss (38.99%). Chen et al. [14] prepared a potato starch-based film incorporating tea polyphenols (TPs); the incorporation of TPs altered the barrier properties of starch membranes against water and oxygen, and markedly slowed down the decrease in weight, hardness, and chewiness of blueberries. Marquez et al. [15] developed a transglutaminase-crosslinked whey protein/pectin edible film, and freshly cut apples were wrapped with it and studied for quality changes. The weight loss of these apples was reduced from 9.5% to 2%, and CFU from 175 CFU/g to 60 CFU/g after 10 days compared to those in the control group. Sarak et al. [16] prepared a film coating based on native starch and a cationic starch blend. This composite film produced the highest elongation at break (399%), which was approximately 11 and 3 times higher than those of N and C films, respectively, and was the best at mitigating mango weight loss (9%).

Pullulan, a polysaccharide consisting of repeating units of maltotriose residues, is produced from starch by the yeast-like fungus *Aureobasidium pullulans* and has been used as a carrier for oral, nasal, and lung transmucosal drug delivery systems. Pullulan exhibits excellent film-forming ability, gas barrier performance, and natural decomposability, making it an optimal material for fruit preservation. Shah et al. [17] altered pullulan via esterification with *n*-octene succinic anhydride and subsequently employed it as a coating to preserve sapota fruits. The water vapor permeability (WVP) of esterified pullulan (degree of substitution: 0.0604) was approximately 30% lower than that of the unmodified pullulan coating. Moreover, this coating delayed the ripening and senescence of sapota fruits. Kou et al. [18] studied the effects of edible coatings (2% CaCl_2_, 1% chitosan, and 1% pullulan) on the nutrient content and antioxidant activity of jujubes; compared with the control fruits, the coated jujubes showed significantly delayed senescence. Chu et al. [19] evaluated the effects of a pullulan coating incorporated with a cinnamon essential oil nanoemulsion on the shelf life and senescence of fresh strawberries during storage; the strawberries coated with the pullulan–cinnamon essential oil nanoemulsion showed markedly lower losses in fruit mass, firmness, TSSs, and TAs after 6-day storage. Tian et al. [20] developed an effective pullulan-based active coating with incorporated food additives that reduced soft rot and extended the shelf life of cold-stored kiwifruits. The fruit decay rate when the pullulan coating contained 10 g/L potassium metabisulfite was 46% lower than that of the control group (without potassium metabisulfite) and the fruit quality was maintained at the end of the shelf life.

Therefore, coating technologies offer numerous benefits for fruit preservation. However, air-drying is typically required to form a film. If the film is damaged or incomplete, its effectiveness in terms of fruit preservation is reduced. In addition, larger fruits require more coating material, resulting in higher costs, and special shapes may lead to problems such as film porosity. Therefore, an urgent need exists for developing safe, convenient, low-cost, and environmentally friendly fruit preservation technologies.

To bridge this gap, in this study, we aimed to develop a simple and effective method for producing pullulan-based packaging paper to enhance the shelf lives of fruits. Using Pul TP Vc as a coating for cling paper allows for direct contact between coating and fruit, ensuring consumption safety. Tea crop-derived TP is chosen as a bacteriostatic agent, while Vc from fruits and vegetables serves as an antioxidant. This will not have a negative effect on those who consume it. First, a composite coating of pullulan with TP and/or Vc was prepared, as TP is a polyphenolic substance extracted from tea and exerts strong, broad-spectrum antibacterial effects [21] and Vc exhibits antioxidant activity [22]. The pullulan-based coating was then applied to base paper using a surface-coating technique. The coated paper was subsequently dried to produce pullulan-based packaging paper. Functional and property analyses confirmed that pullulan effectively acted as a gas barrier by forming a thin film on the paper surface. We further examined the effects of the coating on the physical strength of the paper and the effectiveness of the packaging paper in preserving fruits, with grapes serving as a model fruit. The results of this study could provide valuable insights regarding the application of pullulan-based paper in extending the shelf life of fruits.

## 2. Results and Discussion

### 2.1. Micromorphology of the Packaging Papers

**Figure 1:**
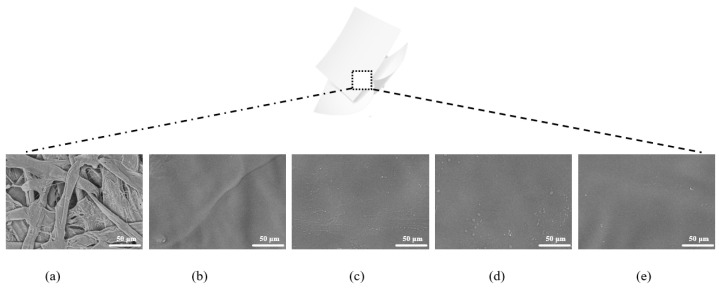
Scanning electron microscopy images of the different types of paper samples. (**a**) Base paper. (**b**) Paper coated with pullulan polysaccharide molecules. (**c**) Paper coated with pullulan and vitamin C. (**d**) Paper coated with pullulan and tea polyphenol. (**e**) Paper coated with pullulan, tea polyphenol, and vitamin C. Panels (**b**–**e**) show paper samples with a coating thickness of 15 ± 0.2 μm.

The microstructures of the paper samples are shown in Figure 1. The surface of the base paper was rough, the fibers were disorderly intertwined, and numerous pores were present between the fibers (Figure 1a). These pores serve as channels that enable the diffusion of gases [23]. By contrast, the pullulan-based paper samples, with or without Vc and/or TP, had flat and smooth surfaces, with no pores between the fibers (Figure 1b–e).

### 2.2. Oxygen- and Water Vapor-Barrier Properties of Different Types of Paper Samples

**Figure 2:**
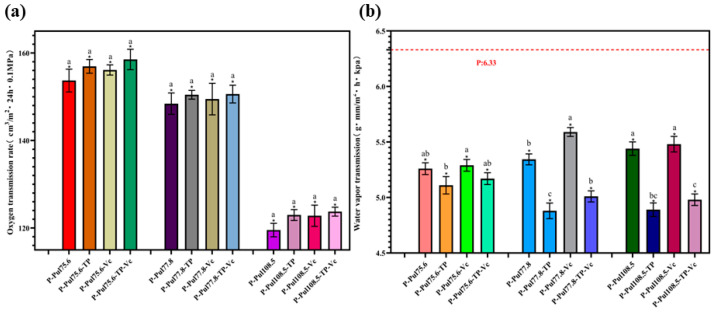
Oxygen- and water vapor-barrier properties of the different types of paper samples with a coating thicknesses of 15 ± 0.2 μm. (**a**) Oxygen transmission rates of paper samples with different coatings. (**b**) Water vapor transmission rates of paper samples with different coatings. P, base paper; Pul, pullulan; TP, tea polyphenol; Vc, vitamin C. Pullulan was used at three different viscosities: 75.6, 77.8, and 108.5 mPa·s. *, *p* < 0.05 compared to the control group, identical letters, *p* > 0.05, differing in letters *p* < 0.05, comparison within group.

The oxygen transmission rate (OTR) was used to characterize the oxygen-barrier properties of different types of papers, whereas the WVTR was used to assess their water vapor-barrier properties. Figure 2 shows the effects of different coatings on the oxygen- and water vapor-barrier properties of the paper samples. The coating treatment significantly improved the oxygen-barrier properties of the cling film (*p* < 0.05). Pullulan was used at three different viscosities: 75.6, 77.8, and 108.5 centipoises (mPa·s). The base paper exhibited almost no barrier to oxygen, whereas the oxygen-barrier properties of the pullulan-based papers increased with increasing viscosity; the paper coated with 108.5 mPa·s pullulan had the lowest OTR (124 cm^3^ m^−2^·24 h·0.1 MPa). These results demonstrated that increasing the standard viscosity of pullulan (which corresponds to a higher molecular weight) improved the film-forming properties and provided a stronger barrier effect against oxygen. Adding TP and/or Vc to the coating material can compromise the oxygen-barrier property of pullulan-based papers. The abundance of hydroxyl groups in pullulan may also contribute to this phenomenon. For example, in a solution, pullulan molecules exhibit intermolecular interactions such as hydrogen bonding and electrostatic forces, which lead to the formation of a distinct network structure [24]. As the solution dries, the gradual evaporation of water results in increased interactions between the pullulan molecules, leading to the formation of a densely interconnected network structure. Simultaneously, the network structure of the film effectively immobilizes the pullulan molecules, resulting in the formation of a stable film. TP and Vc are reducing compounds that can react with the oxygen atoms in pullulan, destroying its structure and weakening the interactions between different component molecules [25], which can reduce the film density. Furthermore, adding TP and/or Vc to the pullulan solution can weaken the cross-linking effect of the membrane or affect the solvent system, thus lowering the oxygen-barrier property.

Figure 2b shows that the water vapor-barrier effect of the base paper was relatively weak (WVTR: 6.33 g mm/m^2^·h·kPa). The coating treatment significantly improved the water vapor-barrier ability of the cling paper (*p* < 0.05). The pullulan-based papers exhibited lower water vapor-barrier properties owing to the presence of dense, strongly hydrophilic films on the paper surface, which prevented the penetration of water molecules from the film to the papers. Adding TP to the pullulan coating reduced the WVTR, possibly due to the strong hydrophobicity of TP, which increased the resistance of the coated paper to water vapor. However, including Vc negatively impacted the water vapor-barrier because Vc is highly water-soluble, making the membrane of the pullulan–Vc coating more permeable to water molecules and thereby compromising its barrier performance.

Accordingly, pullulan-based papers offer key advantages in terms of their exceptional oxygen- and water vapor-barrier properties. This feature is crucial for reducing the exposure of fruits to oxygen, minimizing fruit dehydration, and ultimately extending the shelf lives of fruits.

### 2.3. Free Radical-Scavenging Abilities and Antibacterial Properties of Different Types of Paper Samples

**Figure 3:**
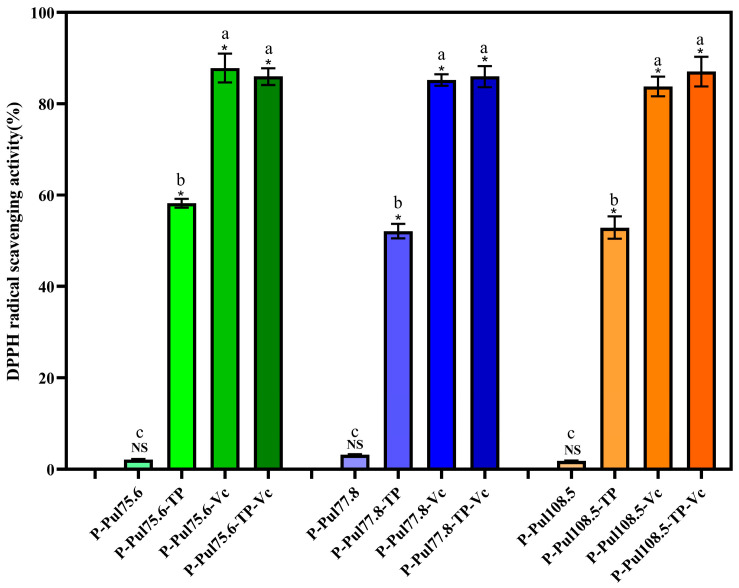
2,2-Biphenyl-1-picrylhydrazyl (DPPH)-scavenging rate of the different paper samples. P, base paper; Pul, pullulan; TP, tea polyphenol; Vc, vitamin C. Pullulan was used at three different viscosities: 75.6, 77.8, and 108.5 mPa·s. NS, *p* > 0.05, *, *p* < 0.05 compared to the control group, identical letters, *p* > 0.05, differing in letters *p* < 0.05, comparison within group.

We used 2,2-biphenyl-1-picrylhydrazyl (DPPH) solution to determine the antioxidant capacities of the tested materials as an indicator of their fruit-preserving potential. Figure 3 illustrates the effect of the coating on the DPPH free radical-scavenging ability of different types of paper samples. The addition of TP and Vc significantly improved the oxidation resistance of the coating (*p* < 0.05). Raw paper demonstrates little or no antioxidant capacity. The base paper and paper coated with pullulan alone showed limited antioxidant activities, with a maximum scavenging rate of 1.06% and 3.2%, respectively; there was no significant effect of the viscosities of the different pullulan preparations on the radical-scavenging activity. However, the pullulan–TP and pullulan–Vc papers showed significantly greater antioxidant activities, with a maximum DPPH radical-scavenging rate of 58.20% and over 87%, respectively. The addition of TP improves the antioxidant capacity of the composite membrane mainly because of the free radical-bursting ability of the hydroxyl groups on the TP molecule. With the addition of Vc, the oxidation resistance of the composite coating is further enhanced because Vc directly interacts with oxidants, which converts oxidized glutathione to reduced glutathione, exerting an antioxidant effect. The antioxidant activity of the pullulan–TP–Vc paper was similar to that of the pullulan–Vc paper. Overall, these results demonstrated that the combination of pullulan with TP and/or Vc showed strong antioxidant properties with the potential to prevent fruit oxidation.

**Figure 4:**
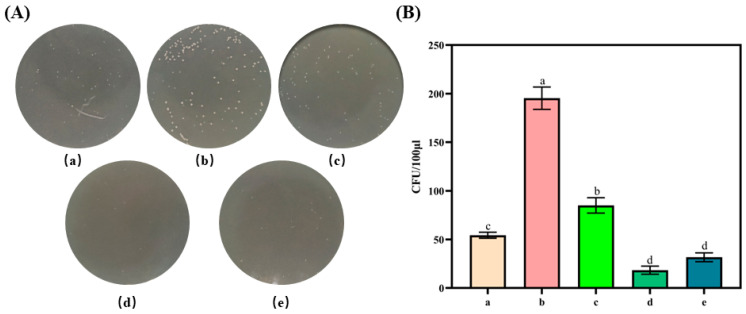
Antimicrobial properties of the different types of paper samples. (**A**) *E. coli* colony, (**B**) The CFU of *E. coli*. (**a**) Base paper. (**b**) Pullulan-coated paper. (**c**) Pullulan–vitamin C-coated paper. (**d**) Pullulan–tea polyphenol-coated paper. (**e**) Pullulan–tea polyphenol–vitamin C-coated paper. Identical letters, *p* > 0.05, differing in letters *p* < 0.05, comparison within group.

Fruit deterioration is largely attributable to the proliferation of microorganisms, including bacteria, fungi, and mold; thus, inhibiting microbial growth during storage is crucial for prolonging the shelf lives of fruits. Accordingly, we further assessed the antibacterial efficacies of different packaging papers using *E. coli* as a representative food-spoiling bacterium. Figure 4 shows the significant effect of coating on the growth in *E. coli* (*p* < 0.05). The CFU of *E. coli* was also determined and the results are shown in Figure 4B. In contrast to the base paper, the pullulan-coated paper stimulated microbial growth. This could be attributed to pullulan providing a substantial carbon source for bacteria. By contrast, the antibacterial efficacy of the pullulan–Vc paper was notably superior to that of the paper coated with pullulan alone, although it was not significantly different from that of the base paper. Moreover, the pullulan–TP paper showed markedly enhanced antibacterial activity, which was primarily attributed to the phenolic hydroxyl groups and benzene rings present in TP that can disrupt the structure of bacterial cell membranes, leading to potent antibacterial effects. The antibacterial efficacy of the pullulan–Vc–TP paper was marginally lower than that of the pullulan–TP paper.

### 2.4. Mechanical Properties of Different Types of Paper Samples

**Figure 5:**
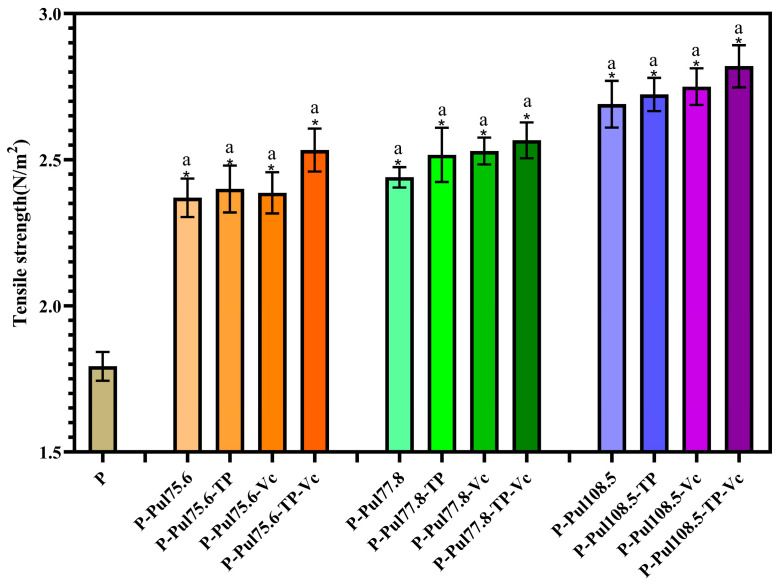
Tensile strengths of the different types of paper samples with a coating thickness of 15 ± 0.2 μm. P, base paper; Pul, pullulan; TP, tea polyphenol; Vc, vitamin C. Pullulan was used at three different viscosities: 75.6, 77.8, and 108.5 mPa·s. *, *p* < 0.05 compared to the control group, identical letters, *p* > 0.05, differing in letters *p* < 0.05, comparison within group.

**Figure 6:**
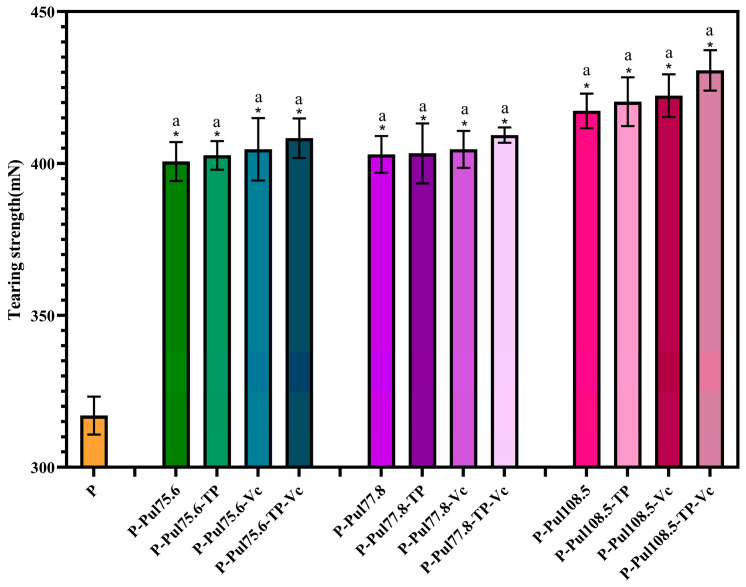
Tearing strengths of the different types of paper with a coating thickness of 15 ± 0.2 μm. P, base paper; Pul, pullulan; TP, tea polyphenol; Vc, vitamin C. Pullulan was used at three different viscosities: 75.6, 77.8, and 108.5 mPa·s. *, *p* < 0.05 compared to the control group, identical letters, *p* > 0.05, differing in letters *p* < 0.05, comparison within group.

Tensile and tearing strengths are important mechanical performance indicators for lightweight packaging paper. Figure 5 and Figure 6 show the influence of the different pullulan-based coatings on these properties.

The coatings significantly increased the tensile strengths at break (*p* < 0.05), with pullulan contributing the most to this improvement, and both parameters increased with increasing standard pullulan viscosities. These results reflect the large number of hydroxyl groups in pullulan molecules, which can form strong hydrogen bonds between the paper fibers [26]. The tearing and tensile strengths of pullulan-based papers were 26.4–35.9% and 32.4–57.5% higher than those of the base paper, respectively. TP and Vc also enhanced the strength of the paper, but the difference was not as substantial as that observed with addition of the pullulan coating; the highest tensile strength (436.67 mN) and tearing strength (2.82 N/m) were found for the paper prepared using Vc, TP, and pullulan at a viscosity of 108.5 mPa·s.

After coating the paper, a significant increase in the paper’s EB was observed. Pul contributes the most to EB, depending on the branched-chain starch collision, winding, and short-term retrogradation among starch chains [27]. The elongation was slightly increased with the addition of TP and Vc to the coating. This is because intermolecular interaction between different molecular chains allowed segmental mobility and sliding of molecular chains against each other. Similar behavior was reported by Kavoosi et al. [28]

### 2.5. Fruit Preservation Performance of Different Types of Paper Samples

#### 2.5.1. Changes in the Weight Loss Rate and Hardness of Grapes

**Figure 7:**
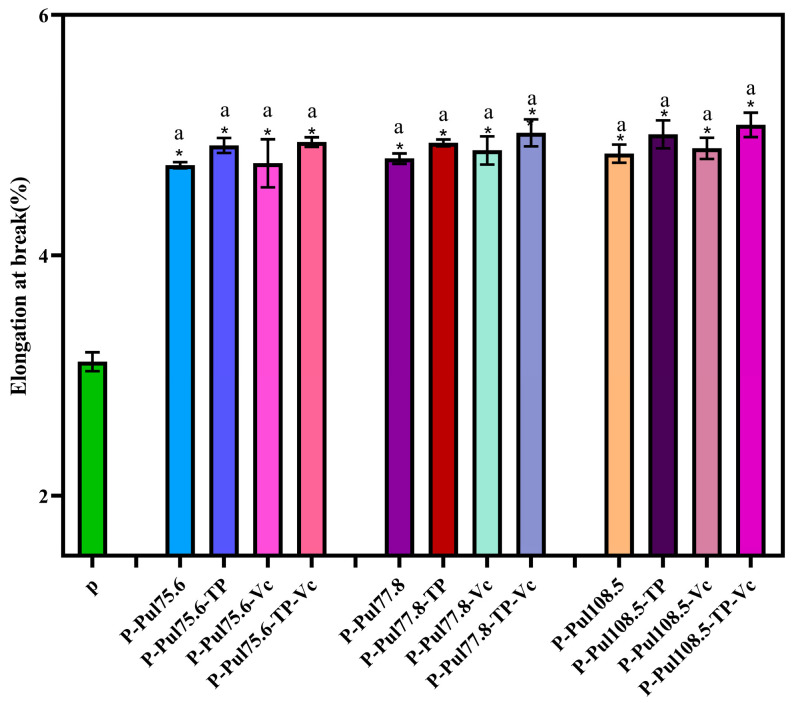
EB of the different types of paper with a coating thickness of 15 ± 0.2 μm. P, base paper; Pul, pullulan; TP, tea polyphenol; Vc, vitamin C. Pullulan was used at three different viscosities: 75.6, 77.8, and 108.5 mPa·s. *, *p* < 0.05 compared to the control group, identical letters, *p* > 0.05, differing in letters *p* < 0.05, comparison within group.

**Figure 8:**
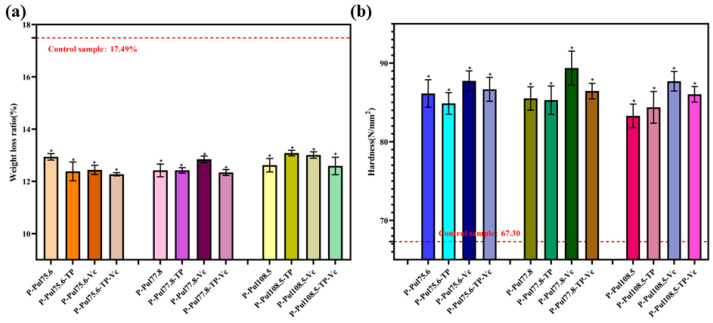
Changes in grape (**a**) weight loss rate and (**b**) hardness. P, base paper; Pul, pullulan; TP, tea polyphenol; Vc, vitamin C. Pullulan was used at three different viscosities: 75.6, 77.8, and 108.5 mPa·s. *, *p* < 0.05 compared to the control group.

After harvest, fruits undergo weight loss and hardness changes owing to differences in respiration, transpiration, and other factors. These changes can negatively affect the appearance and quality of fruits and shorten their shelf lives. Figure 7 shows changes in the weight loss ratio and hardness of grapes packaged in different types of papers. Coated wrappers significantly reduced fruit weight and hardness loss (*p* < 0.05). The weight loss rate of green grapes packaged with various coated papers did not exceed 13.1%, whereas that of green grapes packaged in untreated/base paper (control sample) was 17.49% (Figure 8a). These results suggest that wrapping green grapes in pullulan-based paper can reduce their weight loss. We attributed this finding primarily to the uniform and dense surface of the pullulan-based paper coatings, which effectively reduced the permeability of the paper to oxygen and water vapor. This effectively prevented direct contact between oxygen and the green grapes, thereby inhibiting the respiration of the green grapes and consumption of organic matter. This effect also minimized water and weight loss from green grapes. Among the different types of paper tested, the paper with TP, Vc, and pullulan at a viscosity of 75.6 mPa·s showed the best effect in preventing the weight loss of green grapes, with a weight loss rate of only 12.27%.

Measuring fruit hardness is critical for evaluating ripeness and quality. Increased enzymatic activity and evaporation during storage can decrease fruit hardness. The hardness of all grapes decreased after 10-day storage (Figure 8b). The fruit packaged in the base paper showed the most significant decrease, reaching 67.3 N/mm, whereas grapes wrapped in the pullulan-based papers maintained a hardness of over 84 N/mm^2^.

#### 2.5.2. Changes in TSS and Soluble Protein Contents of Grapes

**Figure 9:**
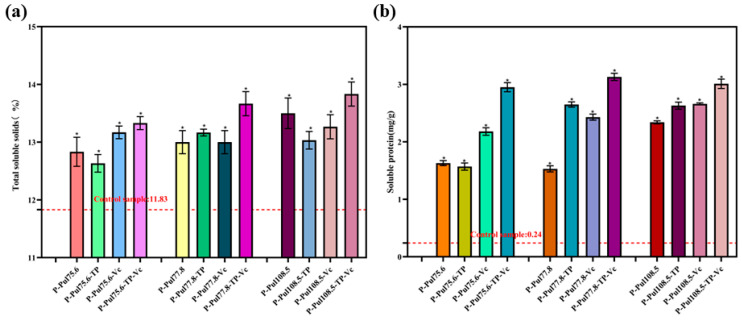
Changes in (**a**) total soluble solids and (**b**) soluble proteins in green grapes 10 days after being wrapped in different types of paper. P, base paper; Pul, pullulan; TP, tea polyphenol; Vc, vitamin C. Pullulan was used at three different viscosities: 75.6, 77.8, and 108.5 mPa·s. *, *p* < 0.05 compared to the control group.

The taste of fruits is directly related to their TSS contents [29]. Although the contents of soluble proteins are low, they still affect the taste and nutritional value of fruits. Figure 9 shows the changes in TSS and soluble protein contents of grapes when stored in different paper samples for 10 days. Coated wrappers significantly reduced the loss of TSS and soluble protein from fruits (*p* < 0.05).

The grapes wrapped in pullulan-based paper had significantly higher TSS and soluble protein contents than those of the control grapes after 10 days of storage (*p* < 0.05). Notably, the loss of TSS in grapes wrapped in pullulan-based paper decreased by 1–2% and the soluble protein content was approximately 6–13-fold higher than that in the control group. These results suggest that pullulan-based paper effectively minimizes the contact between grapes and air, reduces respiration, and prevents the excessive depletion of TSS and soluble proteins [30], thus effectively preserving the fruit quality during storage.

#### 2.5.3. Changes in TA and Ascorbic Acid (AA) Contents of Grapes

**Figure 10:**
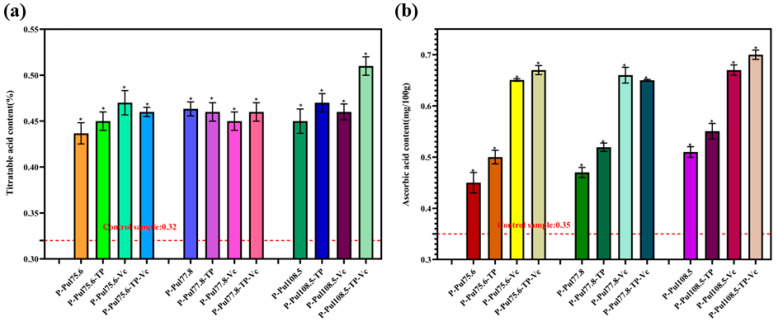
Changes in the (**a**) titratable acid and (**b**) ascorbic acid contents of grapes packaged in different types of paper (control samples: grapes wrapped in untreated/base paper). P, base paper; Pul, pullulan; TP, tea polyphenol; Vc, vitamin C. Pullulan was used at three different viscosities: 75.6, 77.8, and 108.5 mPa·s., *, *p* < 0.05 compared to the control group.

Figure 10 shows the changes in TA and AA contents of base paper-packed grapes and grapes wrapped in the pullulan-coated base papers after 10 days of storage. Coated wrappers significantly reduced the loss of TA and AA from fruits (*p* < 0.05).

Acidity is a crucial indicator of grape flavor and ripeness. During storage, organic acids are metabolized to carbon dioxide and water [31]. Figure 10a shows the TA of grapes after 10-day storage in different papers. Grapes wrapped in pullulan-based papers maintained a significantly higher TA content than the control samples (*p* < 0.05). These data showed that the strong oxygen-barrier effect of pullulan-based paper reduced the oxidation of grapes, slowed their metabolism, and effectively prevented TA degradation [32].

AA (also known as Vc) is an important indicator of the nutritional value of fruits [33]. AA is easily lost during fruit storage because it is relatively unstable and vulnerable to environmental factors such as the presence of light, oxygen, and temperature [34]. AA is oxidized upon exposure to oxygen, leading to its loss. Strong light affects the stability of AA; therefore, fruits are not directly exposed to sunlight during storage. The AA contents in grapes wrapped in pullulan-based papers were significantly higher than those in the control samples (Figure 10b, *p* < 0.05), especially when the pullulan-based paper contained the antioxidant Vc (AA content ≥ 0.65 mg/100 g). This finding suggests that the pullulan-based paper effectively obstructed oxygen and exerted antioxidant effects, thereby creating a low-oxygen storage environment for stored grapes. An oxygen-deficient environment can hinder oxidases [35], thereby slowing the rate of oxidation reactions and inhibiting AA loss in grapes. In addition, the pullulan-based paper could block light during storage and minimize photodamage, which also likely helped to preserve the AA content of grapes.

## 3. Materials and Methods

### 3.1. Materials

Pullulans with viscosities of 75.6, 77.8, and 108.5 mPa·s were provided by Shandong Mimei Biotechnology Co., Ltd. (Weifang, China). TP (97 wt.%), Vc (99 wt.%), bovine serum protein (1 mg/mL), Fehling’s solution A, Fehling’s solution B, anhydrous calcium chloride (96 wt.%), DPPH (98.5%), potassium iodide (99 wt.%), potassium iodate (99.8 wt.%), potassium dihydrogen phosphate (99.5 wt.%), methylene blue (analytical reagent grade), Coomassie brilliant blue G250, and phenolphthalein (biotech grade) were purchased from Shanghai Macklin Biochemical Technology Co. (Shanghai, China). *E. coli* (American Type Culture Collection 11229) were purchased from the Chinese Medicine Culture Preservation Center (Beijing, China). Green grapes (Centennial Seedless; origin, Oakland, CA, USA) and paper (food packaging grade) were bought from a supermarket in Jinan, China. All chemicals used in this study were of analytical grade, and deionized water was used to prepare solutions.

### 3.2. Preparation of Pullulan-Based Packaging Papers

Pullulan polysaccharide preparations with standard viscosities of 75.6, 77.8, and 108.5 mPa·s were added to deionized water (pullulan:water ratio = 15:85, mass ratio). The resulting mixtures were stirred at 70 °C for 1 h to obtain 15 wt.% pullulan solutions. Preliminary experiments showed that 15 wt.% Pul resulted in better adhesion of the coating to the wrapping paper. The TP:Pul ratio of 2:15 (mass ratio) resulted in improved bacteriostatic and physical properties. After obtaining a Vc:Pul ratio of 1:150 (mass ratio), antioxidant properties of the coating no longer changed significantly with increasing Vc content. Therefore, the ratio of Pul:TP:Vc is 15:2:0.1. Therefore, TP and/or Vc were added to the pullulan solutions at a 2:15 and 1:150 (TP/Vc:Pul, mass ratio) ratio, respectively, to obtain four coatings with different compositions. Solutions containing pullulan with either TP, Vc, or both were coated onto base paper (30 g/m^2^) using a coating machine. After drying, different packaging papers with various coatings were obtained with pullulan combined with TP or Vc or coated alone at three different viscosities (75.6, 77.8, or 108.5 mPa·s). The coating thickness was adjusted to 15 ± 0.2 μm because a better oxygen-barrier is achieved at this thickness.

**Figure 11:**
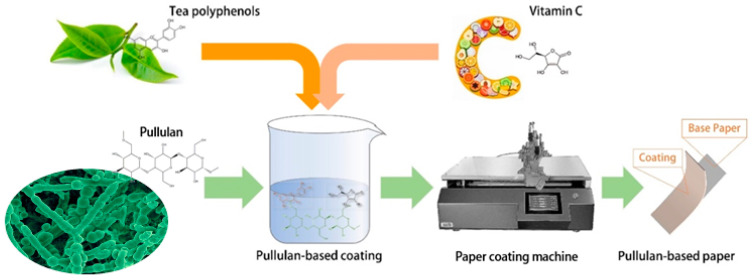
Process used to prepare pullulan-based packaging paper.

The process used to prepare the packaging papers is schematically shown in Figure 11.

### 3.3. Scanning Electron Microscopy

The surface morphologies of the base paper and the paper samples coated with different solutions were observed using a field-emission scanning electron microscope (Hitachi Regulus 8220, Hitachi, Japan). All samples were coated with a thin layer of gold prior to observation.

### 3.4. Mechanical Stability Testing

The paper samples were cut into 50 × 63 mm rectangles and tested for their tearing strength using a tear tester (009; L&W Corporation, Kista, Sweden). The paper samples were cut into 15 × 120 mm rectangles [36], and their tensile strengths were measured using an auto-tensile tester (066; L&W Corporation, Kista, Sweden). Strength tests were performed at least five times for each sample group, and the results were averaged. Tensile properties of packing paper and elongation at break (EB) were determined with a TA-XT texture analyzer (TA-XTPlusC, Stable Micro System, Godalming, UK). Prior to the test, the sample to be tested was cut into a dumbbell strip (h: 50 mm, l: 35 mm) and fixed between two tensile grips. The initial grip separation was set at 20 mm, with a tensile trigger force of 5 g, and the stretching speed was 4.8 mm/min.

### 3.5. Determination of WVTR

Samples were cut into circular pieces of 70 mm diameter and placed in an anhydrous calcium chloride support container for WVP testing. WVP values were determined after 8 h incubation at 37 ± 2 °C with a relative humidity of 90 ± 2%. Equation (1) was used to calculate the WVTR of the paper samples:

(1) $$\mathrm{WVTR}\;(g\;\mathrm{mm}\;m^{-2}\cdot h^{-1}\cdot {\mathrm{kPa}}^{-1})=\frac{\Delta m\;\times \;l}{s\;\times \;t\;\times \;P}$$

Δ*m*: mass change (g), *l*: thicknesses (mm), *s*: square (m^2^), *t*: time (h), *P*: differential pressure (kpa).

### 3.6. Determination of OTR

Samples were cut into circles with a diameter of 74 mm using a disk sampler. The oxygen permeability was tested at 25% and 65% relative humidity using an oxygen permeability tester. The oxygen-barrier experiments were performed at least five times for each sample group and the results were averaged.

### 3.7. Antimicrobial Properties of the Packaging Papers

The gram-negative bacterium *E. coli* (as a representative foodborne pathogen) was used to model the antimicrobial properties of the packaging papers. A 100 μL aliquot of *E. coli* was removed from storage at –20 °C and inoculated into Luria–Bertani (LB) broth. The mixture was incubated in a shaker at 37 °C and 200 rpm for 12 h. The *E. coli* culture was diluted, and a 5 mL inoculum was added to 10-mL centrifuge tubes [37]. The paper samples were cut into circles with diameters of 74 mm, placed in separate 10 mL centrifuge tubes containing *E. coli*, and mixed. The tubes were incubated in a sterile environment for 30 min, after which 100 μL of each bacterial solution was removed, spread onto LB solid culture medium, and incubated at 37 °C to observe colony growth; colonies were counted and expressed as CFU/μL of sample to determine the bacteriostatic properties of the paper samples. Equation (2) was used to calculate *CFU* of *E. coli* of the paper samples:

(2) $$CFU(CFU/\mu L)=\frac{N}{L}$$

*N*: Number of colonies, *L*: Inoculum amount (μL).

### 3.8. Antioxidant Property of the Packaging Papers

The antioxidant properties of the paper samples were measured by performing radical-scavenging tests with DPPH. Briefly, 5 mL of 0.1 mM DPPH solution (dissolved in ethanol) was added to centrifuge tubes. A circular paper sample with a diameter of 74 mm was placed in each tube and shaken thoroughly. Each tube was wrapped in aluminum foil and incubated at 30 °C for 30 min. The absorbance of each solution was measured at 517 nm using an ultraviolet spectrophotometer. Equation (3) was used to calculate the radical-scavenging rates of the paper samples:

(3) $${DPPH}^{*}\;scavenging\;rate\left(\%\right)=\left\{\left(ABS_{0}-ABS_{1}\right)/ABS_{0}\right\}\times 100\%$$

where *ABS*_0_ and *ABS*_1_ represent the absorbance at the initial state and after 1 h of reaction, respectively.

### 3.9. Fruit Property Testing

Before packaging, green grapes of similar shape size and color from the same batch were randomly divided into 13 groups, and the weight was recorded for each group. The grapes were then wrapped in paper with various coatings. The control group consisted of grapes wrapped in the base (untreated) paper. All groups were exposed to the same environmental conditions (30 ± 5 °C, 50 ± 10% RH) for 10 days, after which the changes in the grapes were noted. At least three experimental replicates were performed for each group and the results were averaged.

#### 3.9.1. Weight Loss and Hardness of Fruits

The weights of fruits in the control and packaging paper groups were recorded after 10 days of storage. Equation (4) was used to calculate the weight loss rate of green grapes:

(4) $$W=\frac{m_{2}-m_{1}}{m_{2}}\times 100\%$$

where *W* is the weight loss ratio (%), *m*_1_ is the initial grape weight (g), and *m*_2_ is grape weight on the day of testing.

Fruit firmness is an indicator of fruit maturity and storage quality [38]. Changes in fruit quality can be identified by assessing fruit hardness. An Eidelberg Digital Fruit Hardness Tester (GY-4) was used to measure the hardness of the green grapes. Briefly, peeled grapes were evenly pressed with a 10 mm punch. Equation (5) was then used to calculate the fruit hardness of the grapes:

(5) P=N/S

where *P* is the fruit hardness (N/mm), *N* is the downward pressure peak of the durometer (N), and *S* is the surface under force (mm^2^).

#### 3.9.2. TSS Analysis

The contents of TSSs in grapes were determined using a handheld refractometer. Briefly, 5 g of peeled and pitted green grapes were ground and centrifuged at 25 °C and 4000 rpm for 10 min. Two drops of each supernatant were added to the handheld refractometer to measure the TSS content.

#### 3.9.3. TA Analysis

For each sample, 10 g of peeled and deseeded green grapes was ground and placed in a 100 mL volumetric flask. The flask was then filled with distilled water and allowed to stand for 30 min. The mixture was filtered, and 20 mL of the filtered solution was transferred to a triangular flask containing two drops of 1% phenolphthalein indicator. The solution was titrated with 0.1 m NaOH until it turned pink and remained pink for at least 30 s, indicating that the endpoint had been reached. The amount of NaOH consumed was then recorded. This process was repeated three times. Finally, the *TA* values (wt.%) were calculated using Equation (6):

(6) $$TA=\frac{V\times c\times (V_{1}-V_{0})\times f}{V_{s}\times m}\times 100\%$$

where *V* is the total volume of the sample filtrate solution (mL), *V_s_* is the volume of the filtrate taken during titration (mL), *c* is the NaOH titrant concentration (m), *V*_1_ is the volume of NaOH solution consumed by the titration filtrate (mL), *V*_0_ is the volume of NaOH solution consumed when titrating distilled water (mL), *m* is the sample weight (g), and *f* is the conversion factor (g/m mol). The conversion factor used for green grapes was 0.075.

#### 3.9.4. Soluble Protein

Standard curves were constructed using the protein weight and absorbance values as the x and y coordinates, respectively, and linear regression equations were derived. Peeled and pitted green grapes (2.0 g) were homogenized with 5 mL of deionized water. The resulting homogenate was centrifuged at 4 °C and 12,000 rpm for 20 min to obtain an extract. In a test tube with a stopper, 1.0 mL of the extract (diluted appropriately depending on the protein content) was thoroughly mixed with 5.0 mL Coomassie Brilliant Blue G-250 solution. After a 2 min incubation, the absorbance was measured using the method followed for preparing the standard curve. This process was repeated three times. The soluble protein concentrations (mg/g) were calculated using Equation (7):

(7) $$Sp=\frac{m^{\prime }\times V}{V_{s}\times m\times 1000}$$

where *Sp* is the soluble protein content, *m*′ is the protein weight obtained from the standard curve (µg), *V* is the total volume of the sample extract solution (mL), *V_s_* is the volume of the sample extract solution taken during measurement (mL), and *m* is the sample weight (g).

#### 3.9.5. AA Analysis

Briefly, 0.5 mL KI solution (10 g/L), 2 mL starch solution (5 g/L), 5 mL extract, and 2.5 mL distilled water were combined in a triangular flask. The mixture was titrated with KIO_3_ solution to the titration endpoint. The volume of KIO_3_ solution consumed was recorded, and the experiment was repeated three times. Additionally, 5.0 mL of 2% HCl solution was used to titrate the blank with the same titration method. The *AA* content (mg/100 g) of each sample was calculated using Equation (8):

(8) $$AA=\frac{V\times (V_{1}-V_{0})\times 0.088}{V_{s}\times m}\times 100$$

where *V* is the total volume of sample extract solution (mL), *V*_1_ is the volume of KIO_3_ solution consumed during sample titration (mL), *V*_0_ is the volume of KIO_3_ solution consumed during blank titration (mL), 0.088 is the AA weight equivalent to 1 mL of KIO_3_ solution with a concentration of 1 m mol/L (mg), *V_s_* is the volume of sample solution taken during titration (mL), and *m* is the sample weight (g).

### 3.10. Data Analysis

Each experiment was performed at least three times, and the results were averaged. All graphs were created using OriginPro 2023 software (OriginLab, Inc., Northampton, MA, USA). Data were analyzed using IBM SPSS Statistics 25 software. Significant difference was set at *p* < 0.05.

## 4. Conclusions

The study yielded successful results in preparing Pul, TP, and Vc fruit freshness wrapping paper using the coating method. The study examined the impact of these coatings on the physical, barrier, and functional properties of the original packaging paper. The results revealed that Pul alone enhanced the physical and barrier properties of the paper. Incorporating TP into the coating further improved these properties. This enhancement was attributed to the interaction between Pul and polyphenolic compounds, resulting in strengthened hydrogen bonds and increased coating density, thereby imparting antibacterial and antioxidant properties to the packaging paper. The addition of Vc further boosted the antioxidant activity. Compared to the original packaging paper, Pul-coated paper effectively maintained the hardness, weight, total acidity (TA), and total soluble solids (TSS) of green grapes. Moreover, Pul-coated paper proved more practical for fruit preservation compared with existing coatings, eliminating the need for air- or other drying methods. These findings suggest that Pul-coated packaging paper can significantly prolong the shelf life of fruits, offering a promising option for agricultural production.

## Data Availability

The datasets generated for this study are available upon reasonable request from the corresponding author.
